# Von-Willebrand Disease Presenting as Intractable Epistaxis after Nasal Polypectomy

**DOI:** 10.1155/2014/902071

**Published:** 2014-08-25

**Authors:** Jeong Jin Park, Chang-Hoon Kim, Jeung-Gweon Lee, Hyung-Ju Cho

**Affiliations:** ^1^Department of Otorhinolaryngology, Severance Hospital, Yonsei University College of Medicine, 50 Yonsei-ro, Seodaemoon-gu, Seoul 120-752, Republic of Korea; ^2^The Airway Mucus Institute, Yonsei University College of Medicine, Seoul 120-752, Republic of Korea

## Abstract

Von-Willebrand disease (VWD) is one of the platelet dysfunction disorders that results from a deficiency of Von-Willebrand factor, which is essential for hemostasis. VWD patients typically have normal laboratory results on screening for bleeding disorders. To suspect and diagnose VWD, a careful review of past medical history and laboratory tests is critical. A 59-year-old male patient presented with intractable nasal bleeding after nasal polypectomy. The bleeding was controlled by platelet transfusion, and he was later diagnosed with VWD.

## 1. Introduction

Epistaxis is the most common emergency in ENT departments. To date, many guidelines and algorithms for epistaxis management have been reported, but consensus has not been reached [1, 2]. Most cases of epistaxis can be managed with conservative techniques, but in some cases, cauterization (chemical or electrical) or nasal packing is required. In rare cases, surgical cauterization or sphenopalatine artery ligation or embolization is necessary to control severe epistaxis. We report a case of intractable epistaxis after endoscopic nasal polypectomy, which was later diagnosed as Von-Willebrand disease and managed by platelet transfusion.

## 2. Case Presentation

**Figure 1:**
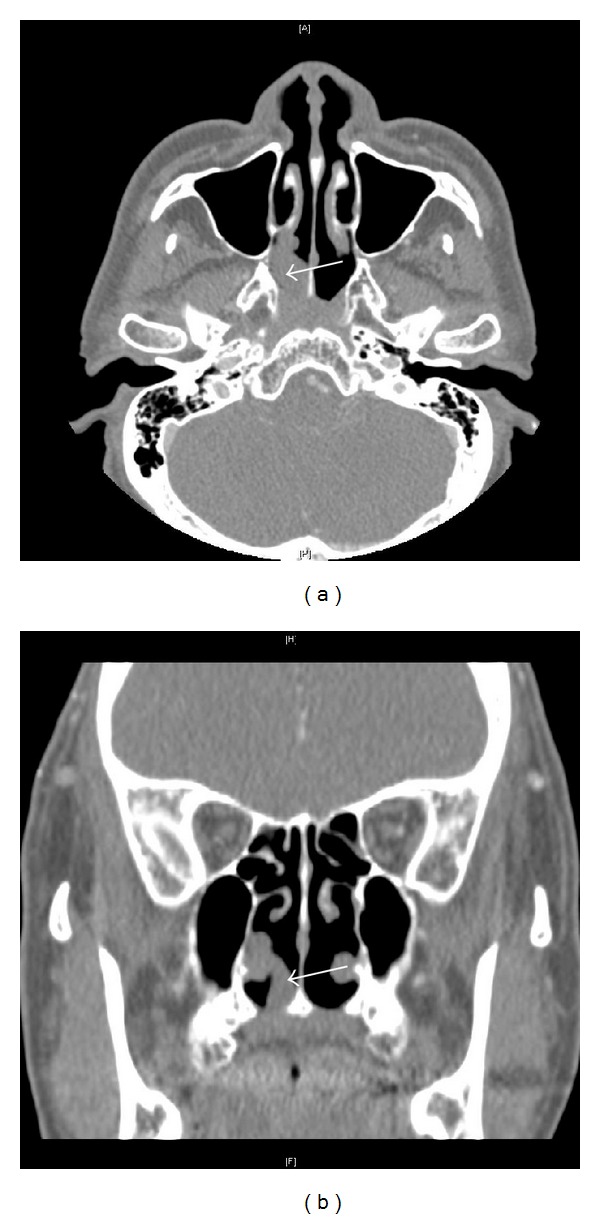
Preoperative paranasal sinus CT. Nasal polyp originating from the posterior portion of the right inferior turbinate is protruding into the choana ((a) axial view, (b) coronal view).

A 59-year-old man underwent endoscopic nasal polypectomy. The polyp, which originated from the right inferior turbinate (Figure 1), was removed easily with minimal bleeding, and nasal packing was performed with Merocel after surgery. The packing was removed the next day, but continuous bleeding from the right nasal cavity developed. To prevent further bleeding, nasal packing was performed again in the right nasal cavity. On the second day after surgery, the packing was removed successfully without further bleeding. However, the man later visited the emergency department due to nasal bleeding. Nasal packing into both nasal cavities was performed again and the bleeding stopped, but rebleeding developed after two days even with intranasal packing.

The laboratory results before nasal polypectomy were all within normal ranges (hemoglobin 9.2 g/dL, platelet count 320 × 10^3^/*μ*L, prothrombin time 9.8 seconds, and activated partial thrombin time 40.8 seconds). When he initially visited the emergency department due to nasal bleeding after polypectomy, hemoglobin was decreased, but platelet count, prothrombin time, and activated partial thrombin time were still in the normal ranges (hemoglobin 6.5 g/dL, platelet 208 × 10^3^/*μ*L, prothrombin time 10.0 seconds, and activated partial thrombin time 39.5 seconds).

**Figure 2:**
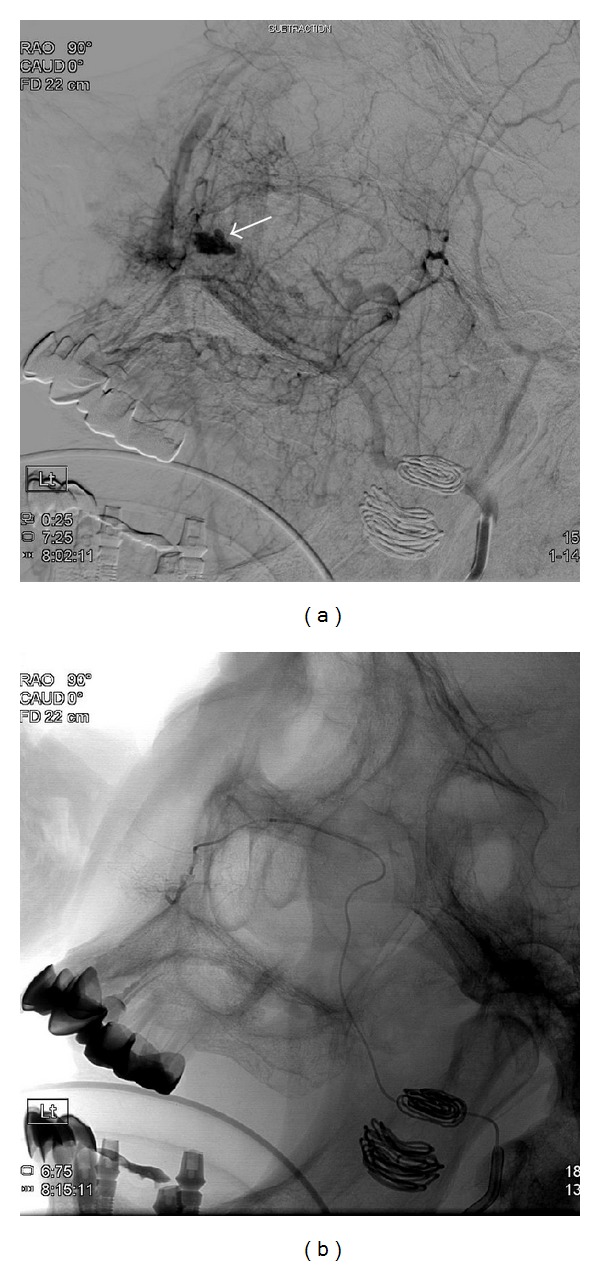
Angiography and arterial embolization. A pseudosac was found at the distal part of the right maxillary arterial branch, but a definite bleeding focus could not be found ((a) preembolization, (b) postembolization).

Emergent surgical cauterization was attempted in the operation room. The bleeding originated from the posterior nasal cavity, but the exact bleeding focus could not be identified and was not controlled by electrical cauterization. To prevent hypovolemic shock because of blood loss, the surgery was stopped, and anterior/posterior packing with Vaseline gauze was performed. The patient was moved to the intensive care unit (ICU) with tracheal intubation to prevent aspiration, and arterial embolization was performed the next day. During angiography, a pseudosac was found at the distal part of the left maxillary arterial branch, but a definite bleeding focus could not be found (Figure 2). Embolization was performed at the pseudosac area, but it was not successful and bleeding persisted. Nasal packing was again performed in the ICU.

The patient did not take any anticoagulative medications and did not have hypertension, diabetes mellitus, or other cardiovascular diseases. However, three years before, he had an episode of hematemesis, which was diagnosed later as gastric angiodysplasia by endoscopy, but a bleeding focus was not found. The hematemesis lasted for several days, and the bleeding eventually stopped spontaneously. Additionally, he had history of bleeding for four days after a dental procedure several years before.

Under suspicion of a coagulative disorder, we consulted a hematologist and performed a coagulation factor assay and testing for platelet function. Factor VIII was 30% and collagen/ADP closure time was prolonged. These results indicated that platelet function was abnormal, and a platelet dysfunction disorder was suspected. Therefore, we conducted platelet transfusion for three days, and the bleeding eventually became sticky and formed a hematoma. On the sixth day after platelet transfusion, the bleeding stopped completely, and we were able to remove tracheal intubation. Red blood cell (RBC) transfusion and albumin supplementation were also necessary. The patient was discharged without any complications after two weeks. To confirm the platelet disorder, further blood testing was performed after platelet transfusion to stabilize the patient. Therefore, the diagnosis with additional confirmatory blood tests was completed ten days after the final platelet transfusion. The laboratory results corresponded to Von-Willebrand disease type IIa. Ristocetin response was decreased, and a low level of VWF multimer was also identified by VWF multimer study.

**Figure 3:**
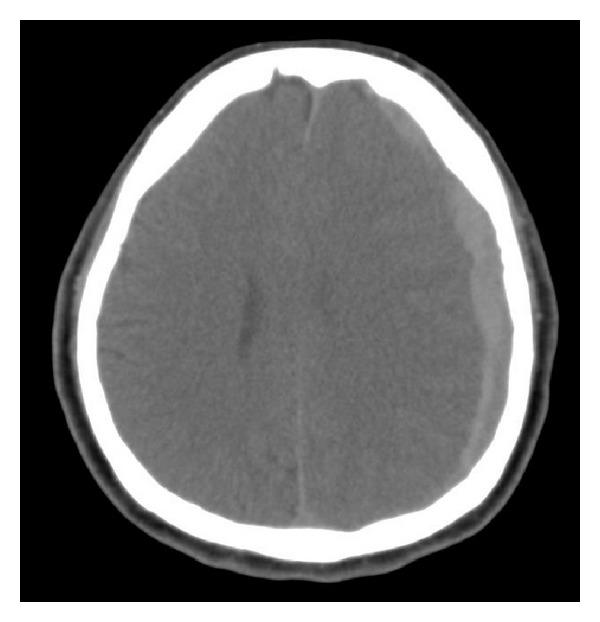
Brain CT (noncontrast), axial view. Subacute subdural hemorrhage was found after head trauma from a fall.

Six months after discharge, the patient fell and developed a subacute subdural hemorrhage (Figure 3). Three neurologic surgeries to remove intracranial hematoma were performed with massive platelet transfusion. His overall function has improved but with the complication of hemiplegia that is being treated with rehabilitation.

## 3. Discussion

Before various surgical procedures, preoperative blood testing such as prothrombin time, activated partial thrombin time, and platelet count is usually mandatory. Furthermore, history taking regarding coagulative disorders, anticoagulative medication history, and bleeding events after simple trauma or procedures is crucial.

In this case, all parameters including PT, aPTT, and platelet count were in normal ranges; however, there was a history of bleeding events after biopsy and a simple dental procedure. The patient was also diagnosed with GI angiodysplasia during an evaluation for hematemesis and hematochezia. Angiodysplasia associated with VWD has been reported for many years [3].

Some coagulative disorders like Von-Willebrand disease, Glanzmann thrombasthenia, Factor VII deficiency, and qualitative platelet disorders present with normal values of PT, aPTT, and platelet count. To differentiate these diseases, both a coagulation factor assay and platelet function testing are required. A low level of Factor VIII and collagen/ADP closure time prolongation indicate dysfunction of platelet aggregation. These results may help to diagnose VWD, but not all cases of VWD show a low level of Factor VIII and prolonged closure time [4]. To confirm VWD in our case, a more specific test was necessary, but platelet transfusion before to reduce bleeding and to stabilize the patient's condition during ICU management was performed before confirmatory lab testing. A similar case was reported about profound bleeding during cleft palate surgery, which was later diagnosed as Von-Willebrand disease. However, that case did not require platelet transfusion since bleeding was controlled during surgery [5].

Von-Willebrand disease (VWD) is the most common inherited bleeding disorder, with a prevalence of about 1% [6]. The pathogenesis of VWD is the deficiency or defect of Von-Willebrand factor (VWF), which is essential for hemostasis. To suspect and diagnose VWD, laboratory testing is important [7]. However, only a small portion of people who have abnormal laboratory test results for VWD present with bleeding symptoms. Therefore, a careful review of past medical and family history is crucial. An unknown explanation of menorrhagia in women and mucocutaneous bleeding during or after invasive procedures should raise suspicion of VWD [7, 8]. Unfortunately, there is no single laboratory test to screen VWD; rather, several specific laboratory studies are required [7]. VWD is typically managed with desmopressin, which enhances the secretion of intrinsic VWF, and by infusion of VWF concentrate. In addition, adjunctive treatment with antifibrinolytic agents (i.e., tranexamic acid) may be helpful.

Even if general laboratory tests such as PT, aPTT, and platelet count show normal results, a coagulative function disorder may still be present. Therefore, it is essential to take a meticulous history of any bleeding event after surgical procedures. Suspicion of a coagulative function disorder and early consultation with a hematologist are essential if a patient has a previous bleeding history and bleeding is not controlled by nasal packing, surgical management, or embolization.
